# NMR Metabolomics of *Arctium lappa* L.*, Taraxacum officinale* and *Melissa officinalis*: A Comparison of Spontaneous and Organic Ecotypes

**DOI:** 10.3390/foods13111642

**Published:** 2024-05-24

**Authors:** Donatella Ambroselli, Fabrizio Masciulli, Enrico Romano, Ruggero Guerrini, Cinzia Ingallina, Mattia Spano, Luisa Mannina

**Affiliations:** 1 Food Chemistry Lab, Department of Chemistry and Technology of Drugs, Sapienza University of Rome, P. le Aldo Moro 5, 00185 Rome, Italy; donatella.ambroselli@uniroma1.it (D.A.); fabrizio.masciulli@uniroma1.it (F.M.); e.romano@uniroma1.it (E.R.); mattia.spano@uniroma1.it (M.S.); luisa.mannina@uniroma1.it (L.M.); 2 NMR-Based Metabolomics Laboratory (NMR Lab), Sapienza University of Rome, Piazzale Aldo Moro 5, 00185 Rome, Italy; 3 Université de Lille, CNRS, UMR 8516—LASIRE—Laboratoire de Spectroscopie Pour les Interactions, la Réactivité et l’Environnement, F-59000 Lille, France; ruggero.guerrini.etu@univ-lille.fr

**Keywords:** officinal plants, pedoclimatic conditions, NMR spectroscopy, metabolomics

## Abstract

Officinal plants are a source of metabolites whose chemical composition depends on pedoclimatic conditions. In this study, the NMR-based approach was applied to investigate the impacts of different altitudes and agronomical practices (Land, Mountain Spontaneous, and Organically Grown Ecotypes, namely LSE, MSE, and OE, respectively) on the metabolite profiles of Burdock root, Dandelion root and aerial part, and Lemon balm aerial part. Sugars, amino acids, organic acids, polyphenols, fatty acids, and other metabolites were identified and quantified in all samples. Some metabolites turned out to be tissue-specific markers. Arginine was found in roots, whereas myo-inositol, galactose, glyceroyldigalactose moiety, pheophytin, and chlorophyll were identified in aerial parts. Caftaric and chicoric acids, 3,5 di-caffeoylquinic acid, and chlorogenic and rosmarinic acids were detected in Dandelion, Burdock and Lemon balm, respectively. The metabolite amount changed significantly according to crop, tissue type, and ecotype. All ecotypes of Burdock had the highest contents of amino acids and the lowest contents of organic acids, whereas an opposite trend was observed in Lemon balm. Dandelion parts contained high levels of carbohydrates, except for the MSE aerial part, which showed the highest content of organic acids. The results provided insights into the chemistry of officinal plants, thus supporting nutraceutical–phytopharmaceutical research.

## 1. Introduction

Officinal plants have been used as a nutrient in traditional cuisines and ethnomedicine throughout history, providing a rich source of nutrients, unique flavours, and cultural significance. Moreover, officinal plants represent a relevant source of bioactive compounds [1] with direct or indirect therapeutic effects [2,3], which are present in specific parts (roots, leaves, and flowers) and also throughout the plant, albeit in different concentrations.

Among several officinal plants, Burdock (*Arctium lappa* L.), Dandelion (*Taraxacum officinale*), and Lemon balm *(Melissa officinalis*) have been used for their edible and medicinal properties [4,5,6,7,8,9].

Burdock, scientific name *Arctium lappa* L., is a biennial herbaceous plant belonging to the Compositae (or Asteraceae) family. The Burdock root is part of the eating habits of Asian populations, who consume fresh roots in tea, salads, and soups after domestic pre-processing [7]. Burdock root contains a large amount of minerals, flavonoids, proteins, phenolic compounds, and polysaccharides, and it is commonly consumed as a vegetable in East Asian cuisine such as stir-fries, soups, and pickles [10,11,12,13]. Due to its nutritional and nutraceutical properties [14], Burdock root is used to produce food supplements such as infusions, extracts, tinctures, and decoctions targeting digestive health, detoxification, and immune support [7,15]. Despite its culinary and medicinal benefits, Burdock may pose risks for individuals allergic to plants in the Asteraceae family. Moreover, its root contains bitter compounds called polyacetylenes, which can be toxic in large amounts [16]. Proper processing and preparation are necessary to mitigate these risks.

Dandelion, scientific name *Taraxacum officinale*, is a leafy perennial herb characterised by a rosette of markedly toothed leaves. Fresh leaves and flowers are used fresh in cuisines as food by being fried or boiled, as well as being consumed fresh in salads., while roots are generally used after being dried as additives in the preparation of jams, teas, and coffees [4,17,18,19]. In particular, it is mainly appreciated as a diuretic and bitter tonic to treat the stomach, liver, and gall bladder. Dandelion hydroalcoholic extracts are also used for their antioxidant and anti-inflammatory effects on cardiovascular risks [20,21], rheumatic complaints, and pain in general [22]. These properties have been attributed to sesquiterpene lactones, polyphenols, phenolic acid derivates, and triterpenoids [5]. Dandelion is “generally recognized as safe” (GRAS) as a food by the U.S. Food and Drug Administration. It can rarely cause allergic reactions, diarrhoea, and gastrointestinal upset [23].

Lemon balm, scientific name *Melissa officinalis*, is an edible officinal herb belonging to the mint family Lamiaceae [24] that commonly grows in the Mediterranean region and western Asia [25]. Lemon balm is mainly used for its aromatic leaves, which have a lemon-like scent and flavour. The leaves are harvested before or during flowering and can be used fresh or dried as food, food ingredients, food supplements, or medicinal agents [26,27].

High amounts of essential oils, triterpenes, and appreciable amounts of caffeic acid-derived polyphenols such as rosmarinic acid and flavonoids have been detected in Lemon balm [28]. Lemon balm is widespread as an herbal drug in extemporaneous use, including extracts and tinctures. In several countries, Lemon balm leaves are used for beverage preparation such as decoctions, infusions, and teas [24] and, therefore, employed in gastrointestinal disorders of nervous origin for treating psycho-vegetative cardiac problems and migraines [29,30,31]. The essential oil and its components are widely employed as flavouring agents (e.g., citral, citronellal, geraniol, linalool) and have been subject to comprehensive safety assessments by various regulatory agencies [32]. The Panel on Food Additives and Nutrient Sources Added to Food (ANS) concludes that due to the lack of an appropriate dossier supporting the use of oregano and Lemon balm extracts as additives, the safety of lemon balm extracts for the proposed uses in eight food categories and use levels of, respectively, 2.0 mg/kg bw/day for women and 2.3 mg/kg bw/day for men cannot be assessed [33]. In the United States, lemon balm is listed as “generally recognized as safe” under 21 CFR Part 182.10 and 182.20 for use as a flavouring agent, adjuvant, or flavour enhancer. The European Medicines Agency (EMA) has evaluated *Melissa officinalis* L., folium for its use as a medicinal agent and has established a traditional use based on sufficient safety data and plausible efficacy. However, there is a lack of conventional clinical safety data [27,34].

To explore their potential benefits and limitations, it is essential to comprehend the diverse roles of these plants, not only in traditional medicine but also as dietary staples or functional foods. It is noteworthy that the chemical and nutritional properties, as well as the physiological and morphological characteristics, of these plants are strongly influenced by environmental factors. In particular, soil and climatic conditions affect the metabolism and productivity of vegetable species [35]. The ability of plants to counter abiotic stress is precisely related to the production of secondary metabolites [36]. Several studies have assessed the effects of soil and climate conditions on crops, such as oilseed fruits [37], vine berries [38], mint [39], and eucalyptus [40], thus providing useful information on the impact of environmental factors and their role in determining chemical profiles.

The literature data regarding the metabolite profiles of Burdock, Lemon balm, and Dandelion have mainly focused on specific bioactive classes, namely polyphenols, terpenes, and lactones, determined using different targeted approaches such as NMR/GC-MS [41], UPLC/QTRAP-MS [42], HPLC-MS [43], and HPLC-DAD [44]. 

Here, for the first time, untargeted NMR-based metabolomics was used to obtain the whole metabolite profiles of Burdock roots, Lemon balm’s aerial parts, and Dandelion roots and aerial parts. This analytical method highlighted the effects of different altitudes and agronomical practices (Spontaneous vs. Organically Grown) on the chemical profile. NMR metabolomics is a recognised suitable approach for identifying and quantifying the primary and secondary metabolites of plants [45,46,47], as well as for the study of metabolic processes [48].

## 2. Materials and Methods

### 2.1. Sampling

Three ecotypes, namely the Land Spontaneous Ecotype (LSE), Mountain Spontaneous Ecotype (MSE), and Organic Ecotype (OE) of Dandelion (*Taraxacum officinale*), Lemon balm (*Melissa officinalis*), and Burdock (*Arctium lappa* L.), were provided by “Fibreno Officinali”, having been collected in Isola del Liri at 150 asl (41°41′ N 13°34′ E, Italy) and Collepardo at 800 asl (41°46′ N 13°22′ E, Italy; Figure S1, Table 1).

Dandelion and Burdock’s ecotypes were harvested in autumn (2022), whereas the Lemon balm ecotype was harvested in spring (2022) at the complete maturity of the plants. The cultivation soil was composed of a mixture of clay and sand. The environmental growing conditions during the harvesting year are shown in Table S1.

Plant collection involved the gathering of specific sections: roots for Burdock, both roots and aerial parts for Dandelion, and aerial parts for Lemon balm. 

Samples were thoroughly washed to remove impurities and freeze-dried (Buchi Lyovapor L-200, Flawil, Switzerland) for three days at −55 °C and 2 × 10^4^ Pa until complete water loss. Afterwards, each sample was blended using a knife mill and stored at −80 °C until extraction.

### 2.2. Chemicals

Methanol (CH_3_OH, HPLC-grade), chloroform (CHCl_3,_ HPLC-grade), and distilled water were obtained from Carlo Erba Reagenti (Milan, Italy). EDTA deuterated (98%) was purchased from Cambridge Isotope Laboratories, Inc. (Andover, MA, USA). Monobasic potassium phosphate (KH_2_PO_4_) and dibasic potassium phosphate (K_2_HPO_4_) were purchased from Aldrich-Fluka-Sigma S.r.l. (Milan, Italy).

Deuterated water (D_2_O) 99.97 atom% of deuterium, methanol-D4 99.80 atom% of deuterium (CD_3_OD), and chloroform-D 99.80 atom% of deuterium (CDCl_3_) were purchased from Euriso-Top (Saclay, France). 

3-(trimethylsilyl)-propionic-2,2,3,3-d4 acid sodium salt (TSP) and 3,4,5-Trimethoxybenzaldehyde (TBZ) were purchased from Merck (Milan, Italy). Polyphenol standards (95%) were purchased from Aldrich-Fluka-Sigma S.r.l. (Milan, Italy).

### 2.3. Extraction Procedure for NMR Analysis

Extractions for NMR analysis followed the Bligh–Dyer protocol [49], albeit with modifications. In particular, 200 mg of sample was added with 3 mL of a CH_3_OH/CHCl_3_ mixture (2:1 *v*/*v*) and 0.8 mL of distilled water. The resulting system was sonicated (thermostat ultrasonic bath ARGOLAB DU-100 (Rome, Italy)) at room temperature for 10 min, before adding 1 mL of chloroform and 1 mL of distilled water. The hydroalcoholic and organic phases were finally separated after centrifugation (Eppendorf Centrifuge 5430 R (Milan, Italy)) for 15 min (25 °C, 7830 rpm). The leftover pellets were extracted twice using the same conditions as previously described. The three extractions obtained from each step were combined. The hydroalcoholic and organic fractions were dried via nitrogen flow. Each sample was prepared and analysed in triplicate.

### 2.4. NMR Analysis

The dried hydroalcoholic phase was dissolved in 700 μL of 100 mM phosphate buffer/D_2_O, containing 0.4 mM TSP as an internal standard. The dried CHCl_3_ fraction was dissolved in 700 μL of CDCl_3_/CD_3_OD (2:1 *v*/*v*) mixture.

NMR analyses were carried out on a JEOL JNM-ECZ 600R (JEOL Ltd., Tokyo, Japan) operating at a proton frequency of 600.17 MHz and equipped with a JEOL 5 mm FG/RO DIGITAL AUTOTUNE probe. Spectra processing and signal integration were performed with JEOL Delta software v5.3.1 (JEOL Ltd., Tokyo, Japan). 

The ^1^H spectra of the hydroalcoholic fraction, as shown in Figure S2, were carried out by using the following parameters: 128 scans, residual water signal suppression with a presaturation pulse, a 7.73 s relaxation delay, a 90° pulse of 8.3 μs, 64 k data points, and a 9000 Hz spectral width. The ^1^H spectra of the apolar fraction were acquired by coadding 64 scans with a 7.73 s relaxation delay and using a 90° pulse of 8.3 μs, 64 k data points, and a spectral window width of 9000 Hz.

Homonuclear ^1^H-^1^H TOCSY and heteronuclear ^1^H-^13^C HMBC and ^1^H-^13^C HSQC experiments for both fractions were carried out following the previously reported experimental parameters [50].

Water-soluble metabolites were quantified by integrating the selected signals and normalising against the TSP methyl group signal (0.00 ppm), set to 100, and quantification was expressed as mg/100 g ± SD of the dried sample. 

For organic extract metabolite quantification, the first step was based on the determination of saturated (SFA) and mono-unsaturated fatty acids (MUFA) integral area values, whose signals overlapped with those of other fatty acids. Their content was calculated by applying the following equations, modifying those previously reported for this purpose [49]:

I_MUFA_ = I_TOT UFA_ − 2I_DUFA_ − 1.5I_TUFA_ (1)

I_TOT SFA_ = I_TOT FA_ − I_MUFA_ − I_DUFA_ − I_TUFA_ (2)

I_MUFA_, I_TUFA_, I_DUFA_, I_TOT UFA_, I_TOT SFA_, and I_TOT FA_ are the integral values of mono-unsaturated fatty acids, tri-unsaturated fatty acids, di-unsaturated fatty acids, total unsaturated fatty acids, total saturated fatty acids, and total fatty acids, respectively. Signals in the 5.33–5.35 ppm range, corresponding to double-bound protons, were considered to integrate TOT UFA. Signals in the 2.28–2.30 ppm range corresponding to α-CH_2_ groups of all fatty acids were considered to integrate TOT FA.

Finally, apolar metabolites were quantified using a 1 mM TBZ external standard. Each fatty acid category was expressed using the main molecule of the class: oleic acid for MUFA, linoleic acid for DUFA, linolenic acid for TUFA, and stearic acid for SFA. Results were expressed as mg/100 g ± SD of dried sample.

For the analysis of hydroalcoholic and organic extracts, the one-way (Burdock and Lemon balm data) and two-way (Dandelion data) ANOVA were applied, respectively, followed by Tukey’s multiple comparison test, to show significant differences (*p* < 0.0001) between the samples considered for each metabolite. GraphPad Prism 8.0.2 software was used for this purpose.

### 2.5. Statistical Analysis

Different subsets of data were generated for the hydroalcoholic extracts of each plant, with the main subsets consisting of the parts analysed (root and aerial part) and the ecotype of each plant (LSE, MSE, OE). A table with the metabolite concentration for each plant was reported in Excel (Office 2016) and subsequently imported into MATLAB (2023a, MathWorks^®^, Natick, MA, USA). Data were pre-processed with autoscaling before performing Principal Component Analysis (PCA) to assess any differences between the three ecotypes of each plant and between the plants themselves.

## 3. Results and Discussions

### 3.1. NMR Assignment of Hydroalcoholic and Organic Fractions

The assignments of the ^1^H NMR spectra of Burdock roots’, Dandelion roots and aerial parts’, and Lemon balm aerial parts’ hydroalcoholic fractions were carried out using 2D experiments (^1^H-^1^H TOCSY, ^1^H-^13^C HSQC, ^1^H-^13^C HMBC), with the addition of the reference standards and literature data relative to other vegetable matrices analysed using the same experimental conditions [45,50]. Primary and secondary metabolites identified in Bligh–Dyer hydroalcoholic extract are reported in Table 2. 

In the high-field NMR region (0.8-3.6 ppm), signals of methyl and methylene groups belonging to aliphatic amino acids (leucine, isoleucine, valine, threonine, alanine, arginine, proline, GABA, glutamine, aspartate, and asparagine) and organic acids (acetate, succinate, citrate, and malate) were observed. The mid-frequency region between 3.0 and 5.5 ppm was dominated by intense signals, mainly due to monosaccharides and disaccharides, namely myo-inositol, β-galactose, glucose, and sucrose. In the low-frequency 6.0–9.0 ppm, spectral region signals of aromatic compounds, namely aromatic amino acids (phenylalanine, tryptophan, and tyrosine), formic and fumaric acids, trigonelline, uracil and uridine were identified. The assignment of polyphenols will be discussed in detail in the next section.

The ^1^H NMR spectra of Bligh-Dyer organic extracts, Figure S3, showed the presence of sterols, fatty acids, lipid polar heads, and pigments, Table 3, identified by literature data [49].

#### NMR Identification of Polyphenols

**Figure 1:**
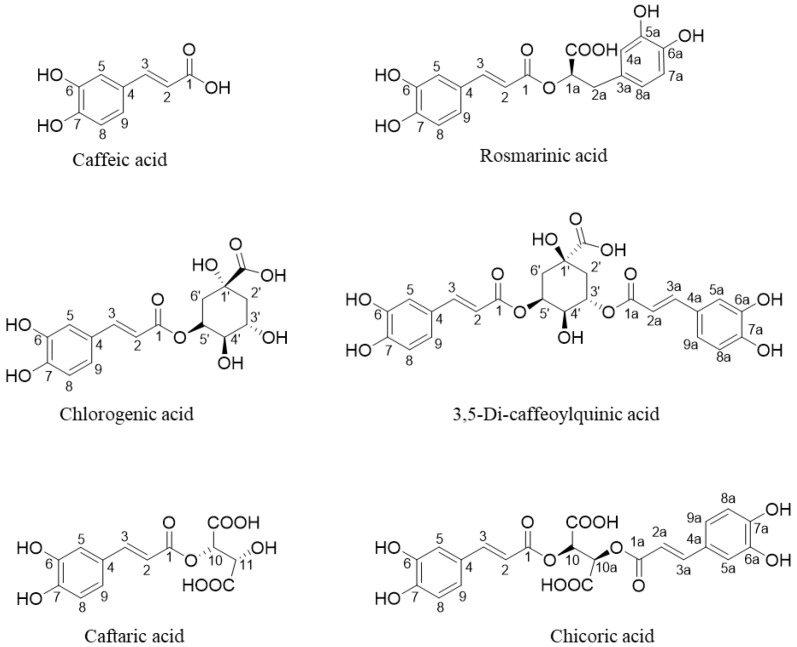
Molecular structures of polyphenols. To make the assignment discussion easier, the numbering of common portions is the same for the compounds.

The Bligh–Dyer hydroalcoholic extracts of Burdock root were characterised by the presence of 3,5-Di-caffeoylquinic acid, as shown in Figure 1 and Table 2.

The ^1^H assignment was achieved through the olefinic proton signals H-2, H-2a, H-3, and H-3a (doublets with *J_trans_* = 16.0 Hz) at 6.13, 6.25, 7.32, and 7.40 ppm, respectively, typical of caffeic moiety α-β unsaturated systems (^13^C signals at 115.7, 116.5, 147.8 and 148.0 ppm, respectively). The two-dimensional ^1^H-^1^H TOCSY experiment allowed for the identification of spin correlations between H-2/H-3 (6.13/7.40 ppm) and H-2a/H-3a (6.25/7.32 ppm) protons. Moreover, two aromatic systems were identified by means of ^1^H and ^1^H-^1^H TOCSY experiments, as well as underlying correlations between protons signals at 6.58 ppm (H-8, d, *J* = 8.3 Hz), 6.70 ppm (H-9, dd, *J* = 8.3; *J* = 1.7), and 7.21 ppm (H-5, d, *J* = 1.7 Hz) and those at 6.66 ppm (H-8a, d, *J* = 8.3 Hz), 6.74 ppm (H-9a, dd, *J* = 8.3 Hz; *J* = 1.7 Hz), and 7.24 ppm (H-5a, d, *J* = 1.7 Hz). Integrating the detected olefinic and aromatic signals revealed the same area value, thus indicating the same molar ratio for each group. This behaviour, observed in all the replicates, suggested the presence of a di-caffeoylquinic compound, whose NMR signals and correlations were in accordance with the literature data [51]. The analysis of quinic moiety in ^1^H and two-dimensional experiments confirmed the relative positions of caffeic acid groups. In particular, two overlapped multiplets at 5.33 and 5.37 ppm were identified due to the quinic moiety protons CH-3′/CH-5′, crossed to CH-2′ and CH_2_-6′ (1.99 ppm; 2.02 ppm) signals in ^1^H-^1^H TOCSY map. Based on the literature data [51], the observed chemical shifts and spin correlations in quinic moiety can only be attributed to 3,5-di-caffeoylquinic acid, thus confirming its presence in Burdock root. 

Chicoric and caftaric acids were identified in Dandelion root and aerial part hydroalcoholic extracts. The presence of chicoric acid, as shown in Figure 1, was assessed through evidence of the spin systems formed by the α-β unsaturated system at 6.49 ppm (H-2 and H-2a, d *J_trans_* = 16.0 Hz) and 7.72 ppm (H-3 and H-3a, d *J_trans_* = 16.0 Hz). ^1^H-^1^H TOCSY and ^1^H-^13^C HSQC experiments also allowed us to observe the aromatic system of chicoric acid evidencing CH-5′ (δ_H_ 6.96, δ_C_ 117.6), CH-6′ (δ_H_ 7.17, δ_C_ 124.2), and CH-2′ (δ_H_ 7.25, δ_C_ 117.6) groups. Moreover, the peculiar signal of chicoric acid, corresponding to (O)CH(COO)-10,10a groups of tartaric moiety, was identified at 5.54 ppm (singlet). The assignment of caftaric acid was carried out considering the peculiar signals of the (O)CH(COO)-10 and (OH)CH(COO)-11 groups of tartaric moiety at 5.31 ppm (d, *J* = 2.2 Hz) and 4.57 ppm (d, *J* = 2.2 Hz), respectively. ^1^H-^1^H TOCSY confirmed the spin correlation between the two signals. Moreover, the α-β unsaturated system of caffeic moiety was detected at 6.41 ppm (d, *J* = 16.0 Hz) and 7.64 ppm (d, *J* = 16.0 Hz). The reported literature data confirmed the identification of both polyphenols and NMR assignments [41].

The ^1^H NMR spectra of Lemon balm hydroalcoholic extracts showed the presence of chlorogenic and rosmarinic acids, as shown in Figure 1. In particular, chlorogenic acid identification was allowed starting from the typical doublets at 7.37 and 6.22 ppm (*J_trans_* = 16.0 Hz) of caffeoyl moiety olefinic protons (CH-3 and CH-2), with short-range carbon correlations observed at 146.8 ppm and 115.8 ppm. Moreover, the presence of signals belonging to the quinic acid moiety was assessed by means of the spin correction observed in the ^1^H-^1^H TOCSY map between CH-4′ (^1^H 3.9 ppm, ^13^C 70.8) and the diastereotopic protons of CH_2_-2′ (^1^H 1.89, 2.10 ppm, ^13^C 41.7 ppm) and CH_2_-6′ (^1^H 1.99, 2.06 ppm, ^13^C 41.7 ppm). Notably, the caffeoyl moiety olefinic protons identified in chlorogenic acid were also detected in rosmarinic acid at the same ppm value. Indeed, doublets at 7.37 and 6.22 ppm were broad due to the overlap of chlorogenic and rosmarinic spin systems. Anyway, rosmarinic acid assignment was based on the presence of the characteristic α proton CH-1a at 5.01 ppm (^13^C 77.5 ppm). In the ^1^H-^1^H TOCSY experiment, the signal showed a correlation with the methylene protons at 2.85 and 2.93 ppm assigned to CH_2_-2a (^13^C 37.9 ppm). These findings agreed with the literature data [52] and NMR spectra of the reference standards.

### 3.2. Characterisation and Comparison among the Three Ecotypes of the Same Species

Hereafter, the metabolite profile of each officinal plant will be discussed separately, and LSE, MSE, and OE will be compared.

#### 3.2.1. Burdock—*Arctium lappa* L.

**Figure 2:**
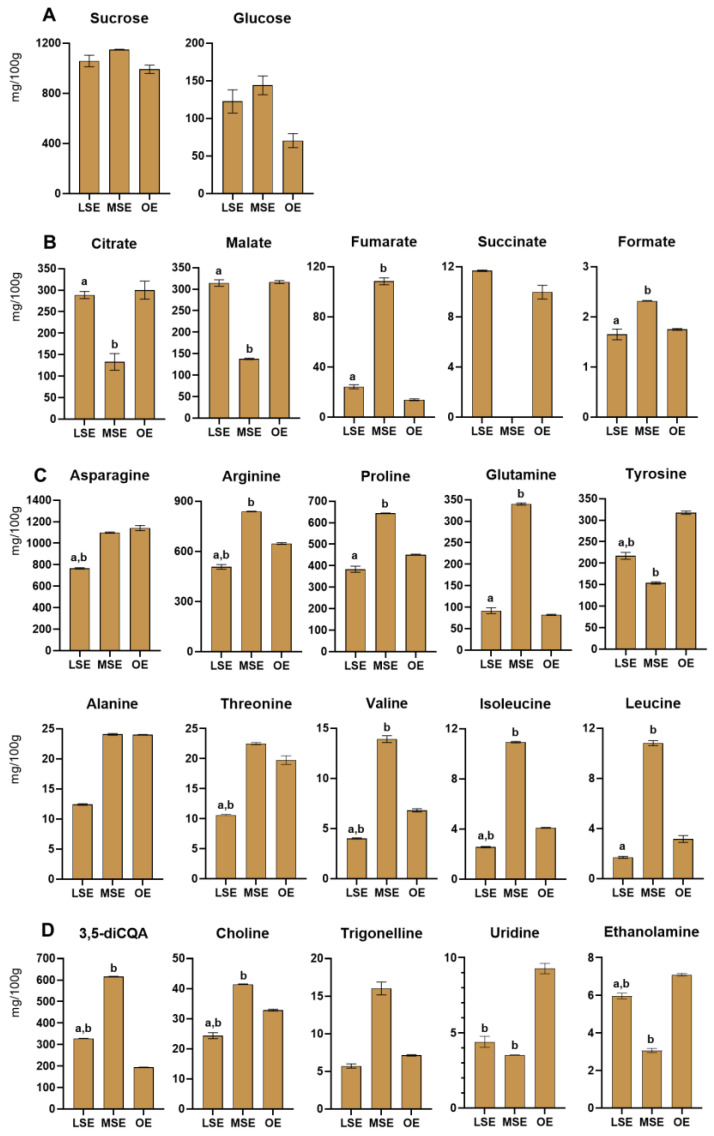
Histograms relative to compounds quantified (mg/100 g of dried sample ± SD) in the Bligh–Dyer hydroalcoholic extracts of Burdock in the Land Spontaneous Ecotype (LSE), Mountain Spontaneous Ecotype (MSE), and Organic Ecotype (OE). (**A**) Sugars, (**B**) organic acids, (**C**) amino acids, and (**D**) other metabolites. One-way ANOVA, followed by Tukey’s multiple comparison test, was applied to underline, among ecotypes, significant differences (*p* < 0.0001) for each metabolite: (a) vs. MSE; (b) vs. OE.

Thirty-six metabolites were identified in the three ecotypes of Burdock root, as shown in Table 2 and Table 3 and quantified in Figure 2. 

Amino acids were the most abundant metabolites among the three ecotypes, followed by carbohydrates and organic acids (Figure S4A). The total contents of sugars were similar in the three ecotypes, with sucrose being the most abundant (Figure 2A). Citrate and malate were measured in the same content range among the organic acids (Figure 2B). Interestingly, fumarate was present in high concentrations in MSE, whereas, on the contrary, succinate was not measured in this ecotype. Regarding the amino acids, MSE extracts were characterised by the highest concentrations of leucine, valine, alanine, proline, threonine, isoleucine, glutamine, and arginine, whereas LSE was characterised by the lowest ones (Figure 2C). Among other compounds, the behaviour observed for polyphenol 3,5-di-caffeoylquinic acid was interesting, with a content of 615 mg/DW in the MSE ecotype, being three times higher in MSE with respect to OE (Figure 2D).

The NMR metabolite characterisation of Burdock root was previously carried out by Jung et al. [42], in which an NMR–metabolomic approach was applied to a methanolic extract of plant root in response to copper stress. Anyway, a more complete NMR assignment concerning organic acids, amino acids, and other metabolites was obtained here. Jung et al. identified only succinate among the organic acids, whereas in the present study, acetate, citrate, formate, fumarate, lactate, and malate were also identified in the hydroalcoholic extracts of Burdock root [42]. Among the amino acids, additional glutamine, tryptophan, and glycine were identified in the ^1^H NMR spectra, while phenylalanine was not detected here. Moreover, other metabolites were found, including choline, ethanolamine, trigonelline, uridine, and the phenolic compound 3,5 di-caffeoylquinic acid.

Regarding liposoluble fraction, histograms were reported in Figure S5. One-way ANOVA was performed, revealing no significant differences (*p* < 0.0001) among the samples. Notably, both Spontaneous ecotypes, with respect to OE, showed a higher level of SFA, whereas the OE extracts were characterised by the highest concentrations of UFAs. Among the latest, DUFA were the main class with respect to TUFA and MUFA. Regarding sterols, β-sitosterol and stigmasterol concentrations were quite similar in all ecotypes. The obtained data were in accordance with the literature in terms of both fatty acids and sterols’ qualitative and quantitative profiles [53]. 

#### 3.2.2. Dandelion—*Taraxacum officinale*

**Figure 3:**
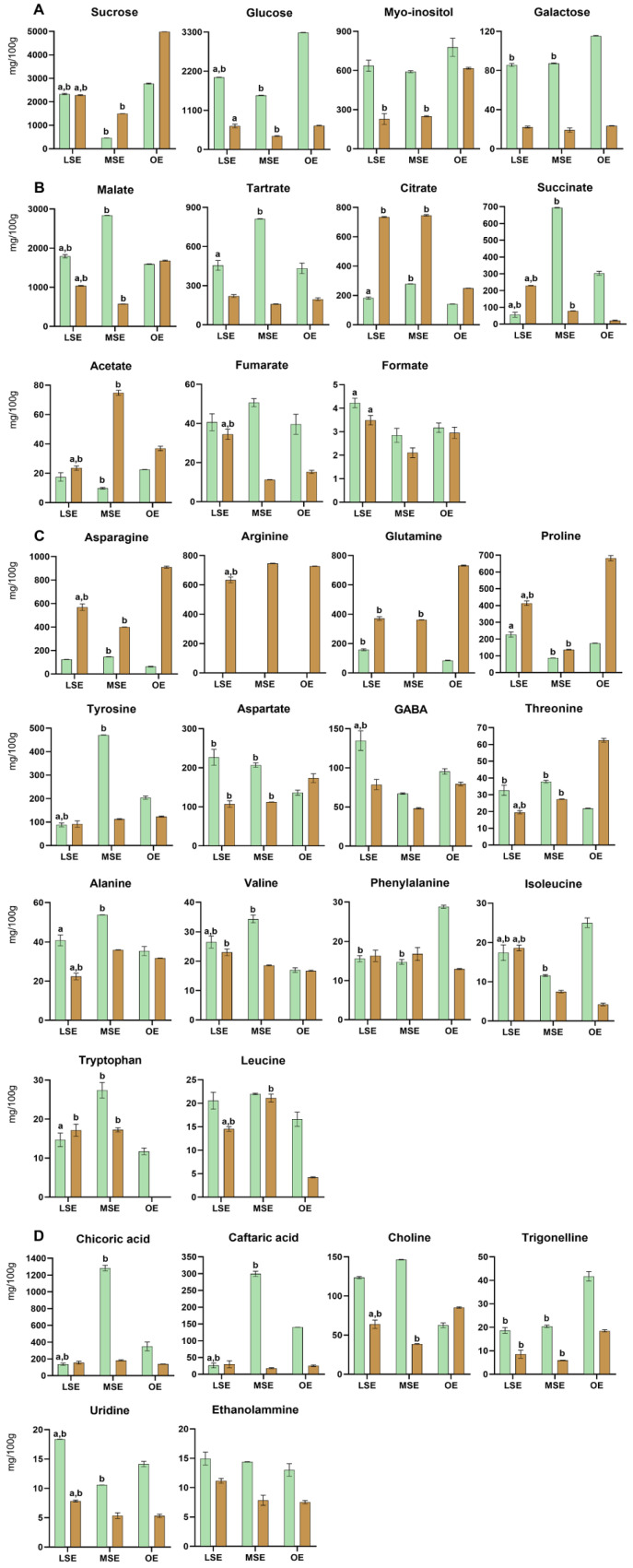
Histograms relative to compounds quantified (mg/100 g of dried sample ± SD) in the Bligh–Dyer hydroalcoholic extracts of Dandelion in Land Spontaneous Ecotype (LSE), Mountain Spontaneous Ecotype (MSE) and Organic Ecotype (OE), comparing the difference between aerial part (green) and root (brown). (**A**) Sugars, (**B**) organic acids, (**C**) amino acids, and (**D**) other metabolites. Two-way ANOVA, followed by Tukey’s multiple comparison test, was applied to underline, among ecotypes, significant differences (*p* < 0.0001) for each metabolite according to the same plant part: (a) vs. MSE; (b) vs. OE.

Forty-five metabolites belonging to different chemical classes were identified in both Dandelion root and aerial part extracts, as shown in Table 2 and Table 3. Histograms of quantified metabolites in the hydroalcoholic phase are shown in Figure 3.

The highest total sugar content was observed for OE ecotypes in both the roots and aerial parts, whereas the lowest was in MSE (Figure S4B). Glucose and sucrose were the most abundant sugars in all ecotypes. Glucose, myo-inositol, and β-galactose were found in higher concentrations in the aerial parts of the three ecotypes, with glucose and β-galactose being three times higher than in the root. On the other hand, root extracts showed a higher concentration of sucrose compared to the aerial parts.

The MSE ecotypes showed the highest total contents of organic acids, with the aerial part extracts particularly enriched. However, few exceptions can be observed for citrate and acetate. In particular, the citrate content was three times higher and comparable in LSE and MSE root extracts. Malate was the most abundant organic acid in all ecotypes, followed by tartrate, citrate, succinate, acetate, fumarate, and formate. 

According to the amino acid profile, the OE root extract showed the highest total content. All identified amino acids were present in both root and aerial part extracts, except for arginine, which was only found in the root. Additionally, glutamine was not detected in the MSE aerial part, and tryptophan was absent from the OE root. Asparagine, proline, and glutamine levels in the root were approximately three times higher than in the aerial part. This is because nitrogen-rich amino acids are used as a storage source of nitrogen in the woody parts of plants, such as the roots [54]. The highest levels of polyphenols (caftaric and chicoric acids) were observed in the MSE aerial parts, with chicoric acid content four-fold higher than in the LSE and OE samples. Choline and trigonelline were abundant metabolites in all ecotypes. 

An NMR-based untargeted metabolomic analysis was previously performed on the methanolic extracts of the Dandelion aerial parts [41]. Here, a more complete assignment of the amino acids profile was achieved, quantifying asparagine, aspartate, threonine, tryptophan, and glutamine. Noteworthy carbohydrates turned out to be the most abundant class of primary metabolites, followed by organic acids and amino acids, in the three ecotypes. In contrast, in Grauso et al. [41], the organic acids were present in higher concentrations than the other classes. These differences are probably due to methodology variability (the extraction solvent’s composition, type of extraction, analytical method), genetic background, and cultivation practices.

The organic Bligh–Dyer extracts of Dandelion (Figure S6) showed that the roots had high levels of SFA, while high levels of MUFA and TUFA mainly characterised the aerial parts, and no SFA was detected in them. Moreover, as expected, pheophytin and chlorophyll pigments were measured only in the aerial parts. Sterol content was quite the same in all the considered samples. Compared with the literature, similar contents of saturated fatty acids were previously measured [41]. A more comprehensive NMR assignment for organic extracts was obtained here.

#### 3.2.3. Lemon Balm—*Melissa officinalis*

**Figure 4:**
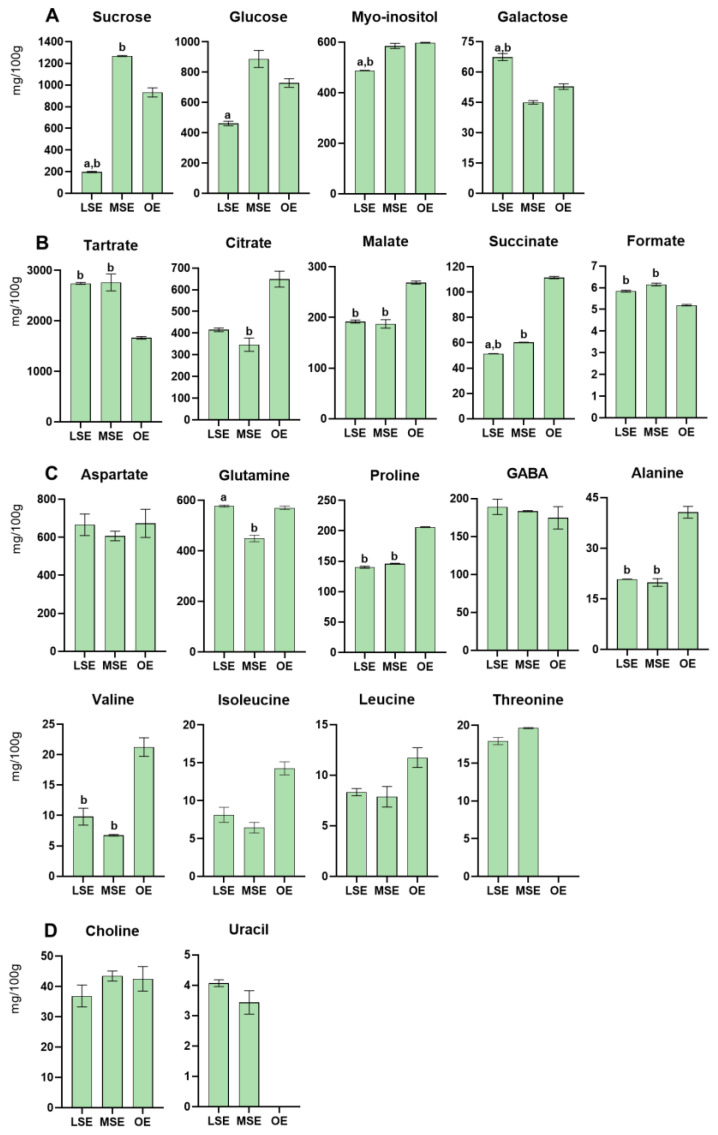
Histograms relative to compounds quantified (mg/100 g of dried sample ± SD) in the Bligh–Dyer hydroalcoholic extracts of Lemon balm in the Land Spontaneous Ecotype (LSE), Mountain Spontaneous Ecotype (MSE), and Organic Ecotype (OE). (**A**) Sugars, (**B**) organic acids, (**C**) amino acids, and (**D**) other metabolites. One-way ANOVA, followed by Tukey’s multiple comparison test, was applied to underline, among ecotypes, significant differences (*p* < 0.0001) for each metabolite: (a) vs. MSE; (b) vs. OE.

Thirty-eight metabolites were detected in Bligh–Dyer hydroalcoholic extracts of LSE, MSE, and OE (Table 2 and Table 3). Histograms resulting from the quantification of water-soluble compounds are reported in Figure 4. 

Lemon balm extracts were rich in organic acids, followed by carbohydrates and amino acids (Figure S4C). The MSE ecotype showed the highest sugar content, whereas the lowest level was observed in LSE. Sucrose and glucose were the most abundant sugars, with the highest concentration in MSE. 

The highest organic acid total content was found in MSE and LSE. Tartrate was the most abundant in the three ecotypes, representing at least 1% of the total dried sample weight in all samples, followed by citrate, malate and succinate. Comparing the three ecotypes, the OE samples had the lowest tartrate contents but the highest citrate, malate, and succinate levels. In contrast, a similar concentration of these metabolites was found in both the Spontaneous LSE and MSE ecotypes. The total amino acid content was comparable in all samples, with the OE ecotype being slightly richer. Aspartate and glutamine were the most abundant amino acids in the three ecotypes, followed by GABA, proline, threonine, alanine, valine, isoleucine, and leucine. The content of each amino acid was similar between the Spontaneous ecotypes, whereas the OE had the highest level, except for threonine, which was not quantified. Choline, uracil, and polyphenols, namely chlorogenic and rosmarinic acids, were also identified. The choline contents in the three ecotypes were comparable, while uracil was not found in the OE. Both rosmarinic and chlorogenic acids were not quantified due to the overlapping signals. 

No literature data concerning exhaustive NMR analyses of Lemon balm have previously been reported, since most studies have exclusively considered the analysis of polyphenols using other analytical techniques (HPLC-DAD, UHPLC-MS) [44,55]. A study on infusion mixtures of different officinal plants, including Lemon balm, using high-resolution ^1^H NMR spectroscopy and multivariate statistical analysis, mentioned the presence of flavonoids and phenols, in particular rosmarinic and chlorogenic acid [56].

Among the liposoluble metabolites reported in Figure S7, the MSE ecotype contained the highest amount of UFAs, especially MUFAs, PC, and DGDG, while the LSE and OE had higher levels of SFAs. However, the highest concentration of TUFA and DUFA was measured in the OE. Sterol content was comparable among the three ecotypes. Phaeophytin and chlorophyll were measured in concentrations lower than 3 mg/100 g, with pheophytin being mainly present in Spontaneous ecotypes. SFA and UFA distributions reported here for Lemon balm aerial parts have also been confirmed by the literature data [57].

### 3.3. Comparison of Burdock, Dandelion and Lemon Balm

The metabolomic profile obtained from the analysis of the selected officinal plants using NMR methodology allowed us to compare the chemical compositions of different ecotypes and tissues and further evaluate the impact of pedoclimatic conditions. The analysis revealed that the qualitative compositions of the most abundant metabolites, including sugars, amino acids, organic acids, and amines, were similar across all samples. However, the levels of these metabolites varied significantly depending on the crop and tissue type. Comparing the total contents of the major metabolites’ classes among the selected samples, the three ecotypes of Burdock roots showed the highest contents of amino acids and the lowest contents of carbohydrates and organic acids, whereas for Dandelion, both the aerial parts and roots were richest in carbohydrates, except for the MSE aerial parts characterised by the highest organic acid total content (4.6 g/100 g of DW). Lemon balm Spontaneous ecotypes were found to have the highest total organic acid contents, exceeding 3 g/100 g of DW (Figure S4). 

Considering the variability in each metabolite, the three officinal plants showed similarities and differences. Branched amino acids were present in smaller amounts, whereas arginine was detected in Dandelion and Burdock roots, being a tissue-specific marker, as well as myo-inositol, galactose, DGG moiety, pheophytin, and chlorophyll, identified in the aerial parts of Lemon balm and Dandelion. Indeed, digalattosyldiacylglycerol stabilises the chloroplast membrane, maintaining its morphology and maximising photosynthesis efficiency to ensure plant survival under abiotic stresses. Pigments such as chlorophyll and pheophytin are involved as electron carrier intermediaries in photosynthesis, which takes place in the leaves. Regarding organic acids, a similar qualitative profile was observed. Tartrate and fumarate were not detected in Burdock and Lemon balm, respectively. Moreover, the comparison of the three officinal plants highlighted the presence of specific secondary metabolites. Burdock, for instance, contained 3,5 di-caffeoylquinic acid, whereas Dandelion contained caftaric and chicoric acids. On the other hand, Lemon balm contained chlorogenic and rosmarinic acids. 

**Figure 5:**
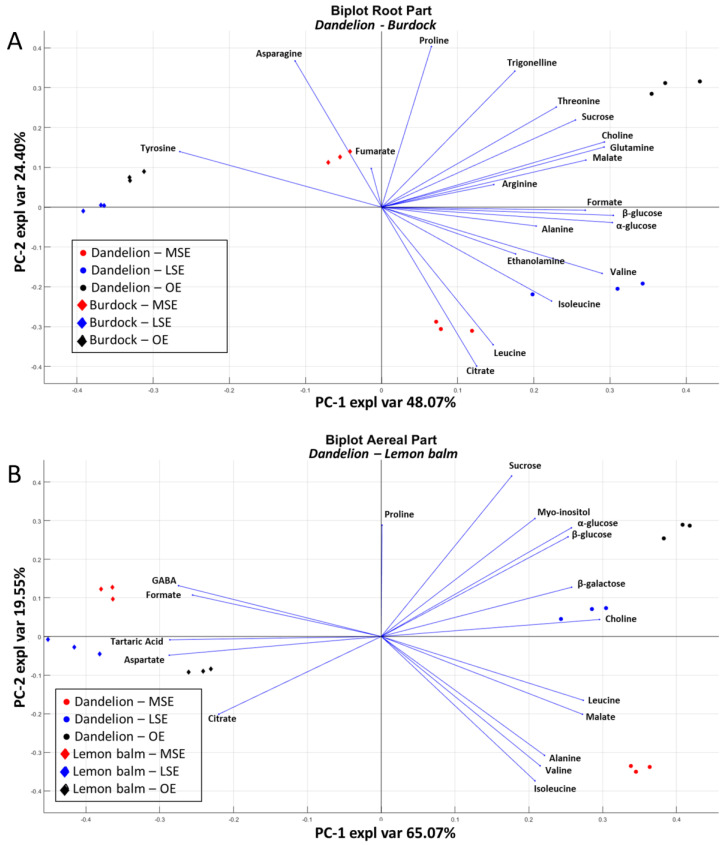
PCA model of (**A**) root parts of Burdock and Dandelion; (**B**) aerial parts of Lemon balm and Dandelion. The points represent the samples, while the lines represent the directions of growth of the plants’ metabolites. In (**A**), the Dandelion samples are represented by circles and the Burdock samples are represented by diamonds. The ecotypes are differentiated by colours: red for MSE, blue for LSE, and black for OE. In (**B**), the Dandelion samples are represented by circles and the Lemon balm samples are represented by diamonds. The ecotypes are differentiated by colours: red for MSE, blue for LSE, and black for OE.

To evaluate the effects of pedoclimatic and genetic factors, the data obtained from the analysis of hydroalcoholic metabolites were subjected to PCA, only considering metabolites always present in the different plants. Two PCA models were made to compare the same parts of different plants, namely one PCA was carried out to compare Burdock and Dandelion roots (Figure 5A), whereas a second one was conducted to compare the aerial parts of Dandelion and Lemon balm (Figure 5B). 

The first PCA model, regarding plant roots, as shown in Figure 5A, accounts for 72.4% of the variability, with separation along PC1 due to genetic basis: positive PC1 values were related to Dandelion and negative ones to Burdock. Dandelion root was shown to be mainly related to higher amounts of all the considered polar metabolites except for tyrosine, asparagine, proline, and fumarate. Moreover, within each considered plant, the obtained model underlined differences among growing conditions. Considering the Dandelion root, the organic ecotype was clearly divided from Spontaneous ones along PC2. On the contrary, in the Burdock root, the clustering occurred between the Mountain Spontaneous ecotype and the remaining ones, along with PC1 negative values. The PCA model of the aerial parts of Dandelion and Lemon balm is reported in Figure 5B, with the first two PCs accounting 84.6% of the variability. Along PC1, samples were separated according to the species, with Lemon balm, with negative PC1 values, being characterised by high levels of GABA, aspartate, and organic acids. Along PC2, a clustering among ecotypes was observed for Dandelion, with MSE being separated from the other ecotypes. For Lemon balm, no clustering among ecotypes was observed.

## 4. Conclusions

NMR metabolomics has allowed us to recognise distinct chemical profiles for each ecotype among the three plants. The untargeted approach demonstrated how each medicinal plant has its own, though in some cases overlapping, chemical profile responsible for its health and nutritional properties. This demonstrates that the pedoclimatic effect significantly impacts the chemical compositions of these plants and highlights the effectiveness of NMR analysis for characterising similar matrices. However, additional sampling is required to establish a stronger correlation among the variables being considered. While PCA was able to take into account all variables in the biplot, further measures are needed to compare different plant species and ensure robustness in the results. From a holistic perspective, it is crucial to complement biological evaluation to obtain comprehensive profiles of ecotypes and plants.

The methodology used here proved its effectiveness in selecting plants with the richest phytochemical profiles. This enhances their value, promotes more conscious consumption, and encourages their use in the nutraceutical, functional food, and phytopharmaceutical industries.

## Figures and Tables

**Table 1:** Description of selected officinal plant ecotypes.

Plant	Harvesting Time	Ecotype	Altitude	Treatment
Burdock (root)	October	Land Spontaneous (LSE)	150 m	No treat, wild grown
		Mountain Spontaneous (MSE)	800 m	No treat, wild grown
		Organic (OE)	150 m	Remotion of other vegetable species from the soil
Dandelion (aerial part and root)	October	Land Spontaneous (LSE)	150 m	No treat, wild grown
		Mountain Spontaneous (MSE)	800 m	No treat, wild grown
		Organic (OE)	150 m	Remotion of other vegetable species from the soil
Lemon Balm (aerial part)	May	Land Spontaneous (LSE)	150 m	No treat, wild grown
		Mountain Spontaneous (MSE)	800 m	No treat, wild grown
		Organic (OE)	150 m	Remotion of other vegetable species from the soil

**Table 2:** Relative assignments of ^1^H and ^13^C NMR signals are of the metabolites identified in the Bligh–Dyer hydroalcoholic extracts of Burdock roots, Dandelion roots and aerial parts, and Lemon balm aerial parts (700 μL of phosphate buffer/D_2_O containing 0.4 mM TSP) are reported. The exponents (^B,D,L^) indicate signals selected for integration. The black dots mark the presence of the metabolite in the officinal plants.

Compound	Assignment	^1^H (ppm)	Multiplicity [*J*(Hz)]	^13^C (ppm)	Burdock	Dandelion	Lemon Balm
*Sugars*							
α-D-Fructofuranose	C-2			105.9	●	●	●
	CH-3	4.11		82.3			
β-D-Fructofuranose	C-2			102.6	●	●	●
	CH-4	4.12		75.6			
β-D-Fructopyranose	C-2			99.3	●	●	●
	CH-3	3.80		68.6			
β-Galactose	CH-1	4.60 ^D,L^	d [7.9]	97.3		●	●
	CH-2	3.51					
	CH-3	3.67					
	CH-4	3.95					
	CH-5	4.05					
	CH-6	3.78					
α-Glucose	CH-1	5.23 ^B,D,L^	d [3.8]	93.3	●	●	●
	CH-2	3.54		72.6			
	CH-3	3.72		73.3			
	CH-4	3.41		70.8			
	CH-5	3.84		72.5			
β-Glucose	CH-1	4.66 ^B,D,L^	d [7.9]	97.0	●	●	●
	CH-2	3.25		75.3			
Inulin	CH-1 (Glc)	5.44		93.9		●	●
	CH-2	3.57		72.1			
	CH-3	3.78		73.6			
	CH-4	3.48		70.2			
	CH-5	3.85		73.4			
	CH_2_-6	3.83		61.1			
	CH_2_-1′ (Fru)	3.75; 3.89					
	CH-3′a	4.21		77.5			
	CH-3′b	4.26					
	CH-4′a	4.05		74.9			
	CH-5′	3.89		82.2			
Myo-inositol	CH-2,5	3.56				●	●
	CH-3,6	3.65					
	CH-4	3.28 ^D,L^		75.0			
Sucrose	CH-1 (Glc)	5.42 ^B,D,L^	d [3.9]	93.1	●	●	●
	CH-2	3.53		71.8			
	CH–3	3.72		73.6			
	CH–4	3.42		70.2			
	CH–5	3.84		73.5			
	C2′ (Fru)			104.8			
	CH–3′	4.23		77.4			
	CH–5′	3.92		82.4			
	CH–4′	3.83		61.2			
*Organic acids*							
Acetate	CH_3_	1.92 ^B,D^	s	24.4	●	●	●
	COO^-^			184.4			
Citrate	α, γ-CH	2.56 ^B,D,L^	d [16.0]	46.0	●	●	●
	α′, γ′-CH	2.68		46.0			
	β-C			73.2			
	1,5-COO^-^			180.2			
	6-COO^-^			183.0			
Formate	HCOO^-^	8.46 ^B,D,L^	s		●	●	●
Fumarate	α, β-CH=CH	6.53 ^B,D^	s	136.7	●	●	
Lactate	β -CH_3_	1.34 ^B^	d [7.1]	21.4	●		
Malate	α-CH	4.31 ^B,D,L^	dd [9.9; 3.1]	71.0	●	●	●
	β-CH	2.68	dd [15.5; 3.1]	43.5			
	β′-CH	2.41	dd [15.5; 9.9]	43.5			
Succinate	α, β-CH_2_	2.42 ^B,D,L^	s	35.2	●	●	●
Tartrate	CH(OH)COO^-^	4.34 ^D,L^	s	75.3		●	●
*Amino acids*							
Alanine	α-CH	3.80		51.0	●	●	●
	β-CH_3_	1.49 ^B,D,L^	d [7.2]	17.3			
	COO^-^			178.6			
Arginine	α-CH	3.77		55.4	●	● *	
	β-CH_2_	1.91	m	28.0			
	γ-CH	1.67 ^B,D^	m	25.3			
	γ′-CH	1.74	m	25.3			
	δ-CH_3_	3.24		41.5			
Asparagine	α-CH	4.00		52.8	●	●	
	β, β′-CH_2_	2.88 ^B^; 2.96 ^D^	dd [7.7; 16.8] dd [4.3; 16.8]	35.6			
	COO^-^			176.5			
Aspartate	β, β′-CH_2_	2.70 ^L^; 2.81 ^D^	dd [3.7; 17.5]	37.0		●	●
	α-CH	3.92		54.0			
	γ-COO^-^			177.3			
GABA	α-CH_2_	2.30 ^D,L^	t [7.4]	35.4		●	●
	β-CH_2_	1.90		24.9			
	γ-CH_2_	3.01	t [7.5]	40.4			
Glutamine	α-CH	3.76		55.3	●	●	●
	β, β′-CH_2_	2.14	m	27.5			
	γ-CH	2.45 ^B,D,L^	m	31.9			
Glycine	α-CH_2_	3.58	s	42.5	●		
Isoleucine	α-CH	1.97		38.0	●	●	●
	β-CH	1.27		29.4			
	γ-CH_3_	1.01 ^B,D,L^	d [7.1]	15.7			
	δ-CH_3_	0.89	d [7.4]				
Leucine	β-CH_2_	1.74		41.0	●	●	●
	γ-CH	1.71					
	δ-CH_3_	0.97 ^B,D,L^	d [6.2]	23.1			
	δ′-CH_3_	0.96		22.1			
Phenylalanine	CH-2,6	7.33	m	130.7		●	
	CH-4	7.38					
	CH-3,5	7.43 ^D^	m	130.6			
Proline	α-CH	4.13 ^L^		62.5	●	●	●
	β, β′-CH_2_	2.07, 2.33		30.0			
	γ-CH_3_	2.01 ^B,D^	m	25.0			
	δ, δ′-CH_3_	3.33, 3.41		47.4			
Threonine	α-CH	3.59		62.3	●	●	●
	γ-CH_3_	1.33 ^B,D,L^	d [6.7]	20.7			
Tyrosine	CH-2, 6 ring	7.22	d [8.2]	132.0	●	●	
	CH-3, 5 ring	6.96 ^B,D^	d [8.5]	117.0			
Tryptophan	CH-4 ring	7.74			●	●	
	CH-5 ring	7.20					
	CH-6 ring	7.29					
	CH-7 ring	7.53 ^B,D^	d [8.1]				
Valine	α-CH	3.60			●	●	●
	β-CH	2.27					
	γ-CH_3_	1.10	d [7.1]	17.7			
	γ′-CH_3_	1.04 ^B,D,L^	d [7.1]	19.0			
*Other compounds*							
Choline	+N(CH_3_)_3_	3.20 ^B,D,L^	s	55.1	●	●	●
Ethanolamine	α, β-CH_2_	3.14 ^B,D^	t [5.2]	42.1	●	●	
Trigonelline	CH-1	9.13 ^B,D^	s		●	●	
	CH-3	4.42	s				
	CH-4	8.09					
Caftaric acid	CH-10	5.31 ^D^	d [2.2]			●	
	CH-11	4.57	d [2.2]				
	CH-2	6.49	d [16.0]	115.4			
	CH-3	7.72	d [16.0]	148.0			
	CH-8 ring	6.96	d [8.2]	117.6			
	CH-9 ring	7.17	dd [2.0; 8.2]	124.2			
	CH-5 ring	7.25	d [2.0]	117.5			
	COO^−^			171.4			
Chicoric acid	CH-10,10a	5.54 ^D^	s	75.9		●	
	CH-2, 2a	6.49	d [16.0]	115.4			
	CH-3, 3a	7.72	d [16.0]	148.0			
	CH-8, 8a ring	6.96	d [8.2]	117.6			
	CH-9, 9a ring	7.17	dd [2.0; 8.2]	124.2			
	CH-5, 5a ring	7.25	d [2.0]	117.5			
	COO^−^			171.4			
Chlorogenic acid	CH-2	6.22	d [16.0]	115.8			●
	CH-3	7.37	d [16.0]	146.8			
	CH_2_-2′	1.89; 2.10	m	41.7			
	CH-4′	3.90	m	70.8			
	CH_2_-6′	1.99; 2.06	m	38.6			
3,5-Di-caffeoylquinic acid	CH-2, 2a	6.13, 6.25 ^B^	d [16.0]	115.7, 116.5	●		
	CH-3, 3a	7.40, 7.32	d [16.0]	148.0, 147.8			
	CH-3′, 5′	5.335.37	m m	72.3 72.9			
	CH_2_-2′, 6′	1.99, 2.02	m	40.0			
	CH-8, 8a ring	6.58, 6.66	d [8.3]	116,9, 117.0			
	CH-9, 9a ring	6.70, 6.74	dd [1.7; 8.3]	123.9, 124.0			
	CH-5, 5a ring	7.21, 7.24	d [1.7]	116.2, 117.0			
Rosmarinic acid	CH-2	6.22	d [16.0]	115.8			●
	CH-3	7.37	d [16.0]	146.8			
	CH-1a	5.01	m	77.5			
	CH_2_-2a	2.85; 2.93	m	37.9			
Uracil	CH	7.84 ^L^	d				●
	CH	5.80	d				
Uridine	CH-6	5.90 ^B^	d [8.1]		●	●	
	CH-5	7.87 ^D^	d [8.1]				
	CH-1′	5.89	d [4.8]				

The exponent ^“B”^ indicates selected signals used for the integration of the Burdock (*A. lappa* L.) metabolites. The exponent ^“D”^ indicates selected signals used for the integration of the Dandelion (*T. officinale*) metabolites. The exponent ^“L”^ indicates selected signals used for the integration of the Lemon balm (*M. officinalis*) metabolites. * Detected only in Dandelion’s radical part.

**Table 3:** Compounds and relative selected signals (ppm) for quantitative analysis in the organic extracts of Burdock root, Dandelion root and aerial part, and Lemon balm aerial part. The black dots mark the presence of the metabolite in the officinal plants.

	ppm	Group	Compounds	Burdock	Dandelion		Lemon Balm
					Root	Leaves	
I_β-Sit_	0.65	CH_3_-18	β-Sitosterol	●	●	●	●
I_Stig_	0.67	CH_3_-18	Stigmasterol	●	●	●	●
I_FA_	2.30	CH_2_-11	Totally fatty acids	●	●	●	●
I_DUFA_	2.73	CH_2_-11	Linoleic acid	●	●	●	●
I_TUFA_	2.77	CH_2_-11,14	Linolenic acid	●	●	●	●
I_PCG_	3.23	N(CH_3_)_3_	Glyceroylphosphatidylcholine	●	●	●	●
I_DGG_	4.87	CH-1	Glyceroyldigalactose			●	●
I_PHEO_	11.15	CH-5	Pheophytin			●	●
I_CHL_	11.13	CH-5	Chlorophyll			●	●

## Data Availability

The original contributions presented in the study are included in the article and Supplementary Materials, further inquiries can be directed to the corresponding author.

## Supplementary Materials

The following supporting information can be downloaded via this link: https://www.mdpi.com/article/10.3390/foods13111642/s1, Figure S1. Burdock root (A), Dandelion aerial part and root (B), and Lemon balm aerial part (C); Figure S2. ^1^H-NMR Spectrum of the hydroalcoholic Bligh–Dyer extract in 100 mM PBS/D_2_O and 0.4 mM TSP. (a) Burdock root, (b) Dandelion root, (c) Dandelion aerial part, and (d) Lemon balm aerial part; Figure S3. ^1^H-NMR Spectrum of the organic Bligh–Dyer extract in CDCl_3_/CD_3_OD (2:1 *v*/*v*) mixture. (a) Burdock root, (b) Dandelion root, (c) Dandelion aerial parts, and (d) Lemon balm aerial part; Figure S4. Histograms resulting from the quantitative NMR analysis of the main compound’s total contents of amino acids (orange), organic acids (light blue) and carbohydrates (yellow) present in the Bligh–Dyer hydroalcoholic extracts of (A) Burdock root, (B) Dandelion aerial part and root, and (C) Lemon balm aerial part in the three ecotypes: Land Spontaneous (LSE), Organic (OE), and Mountain Spontaneous (MSE). Results, expressed as mg/100 g of dried sample, refer to the mean and SD of three replicates; Figure S5. Histograms relative to compounds quantified (mg/100 g of dried sample ± SD) in Bligh–Dyer organic extracts of Burdock in Land Spontaneous (LSE), Mountain Spontaneous (MSE), and Organic (OE); Figure S6. Histograms relative to compounds (mg/100 g dried sample ± SD) in Bligh–Dyer organic extracts of Dandelion’s aerial part (green) and roots (brown) in Land Spontaneous (LSE), Mountain Spontaneous (MSE), and Organic (OE). Two-way ANOVA, followed by Tukey’s multiple comparison test, was applied to underline, among ecotypes, significant differences (*p* < 0.0001) for each metabolite according to the same plant part: (a) vs. MSE; (b) vs. OE; Figure S7. Histograms relative to compounds quantified (mg/100 g of dried sample ± SD) present in Bligh–Dyer organic extracts of Lemon balm in Land Spontaneous (LSE), Mountain Spontaneous (MSE), and Organic (OE). One-way ANOVA, followed by Tukey’s multiple comparison test, was applied to underline, among ecotypes, significant differences (*p* < 0.0001) for each metabolite: (a) vs. MSE; (b) vs. OE; Table S1. Environmental growing conditions in Collepardo and Isola del Liri (Italy).

## Abbreviation

DGG	Digalactosyldiacylglycerol
DUFA	Di-unsaturated fatty acids
GABA	γ-amino-butyric acid
GC-MS	Gas Chromatography–Mass Spectroscopy
^1^H-^13^C	HSQC Heteronuclear single quantum coherence Spectroscopy
^1^H-^13^C	HMBC Heteronuclear Multiple Bond Correlation Spectroscopy
^1^H-^1^H	TOCSY Total Correlation Spectroscopy
HPLC-DAD	High-Performance Liquid Chromatography–Diode Array Detector
HPLC-MS	High-Performance Liquid Chromatography–Mass Spectroscopy
LSE	Land Spontaneous Ecotype
MUFA	Mono-unsaturated fatty acids
MSE	Mountain Spontaneous Ecotype
NMR	Nuclear Magnetic Resonance
OE	Organic Ecotype
PCA	Principal Component Analysis
QTRAP-MS	Mass Spectroscopy
UFA	Unsaturated Fatty Acids
UHPLC-MS	Ultra-High-Performance Liquid Chromatography-Mass Spectroscopy
UPLC	Ultra-Pressure Liquid Chromatography
TBZ	3,4,5-Trimethoxybenzaldehyde
TSP	3-(trimethylsilyl)-propionic-2,2,3,3-d4 acid sodium salt
TUFA	Tri-unsaturated fatty acids
