# Supplementary material for: NMR Metabolomics of Arctium lappa L., Taraxacum officinale and Melissa officinalis: A Comparison of Spontaneous and Organic Ecotypes
Source: Foods. 2024 May 24;13(11):1642. doi: 10.3390/foods13111642 (PMC11171743; doi:10.3390/foods13111642)
Supplement: Supplementary file 1 [file foods-13-01642-s001.zip › foods-3012283-supplementary.pdf]

Supplementary Materials

NMR Metabolomics of *Arctium lappa* L., *Taraxacum officinale* and *Melissa officinalis*: A Comparison of Spontaneous and Organic Ecotypes

Donatella Ambroselli[1,2], Fabrizio Masciulli[1,2], Enrico Romano[1,2], Ruggero Guerrini[3], Cinzia Ingallina[1,2] *, Mattia Spano[1,2], and Luisa Mannina[1]

[1] Food Chemistry Lab, Department of Chemistry and Technology of Drugs, Sapienza University of Rome, P. le Aldo Moro 5, 00185 Rome, Italy; donatella.ambroselli@uniroma1.it, fabrizio.masciulli@uniroma1.it, e.romano@uniroma1.it, mattia.spano@uniroma1.it, luisa.mannina@uniroma1.it

[2] NMR-based Metabolomics Laboratory (NMR Lab), Sapienza University of Rome, Piazzale Aldo Moro 5, 00185 Rome, Italy

[3] Université de Lille, CNRS, UMR 8516—LASIRE—Laboratoire de Spectroscopie Pour les Interactions, la Réactivité et l'Environnement, F-59000 Lille, France; ruggero.guerrini.etu@univ-lille.fr

*Correspondence: cinzia.ingallina@uniroma1.it

Figures

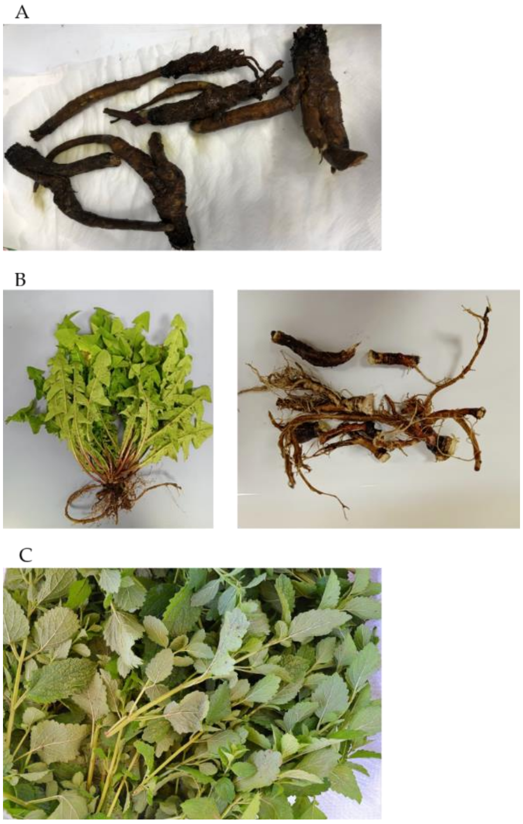

Figure S1. Burdock root (A), Dandelion aerial part and root (B), Lemon balm aerial part (C)

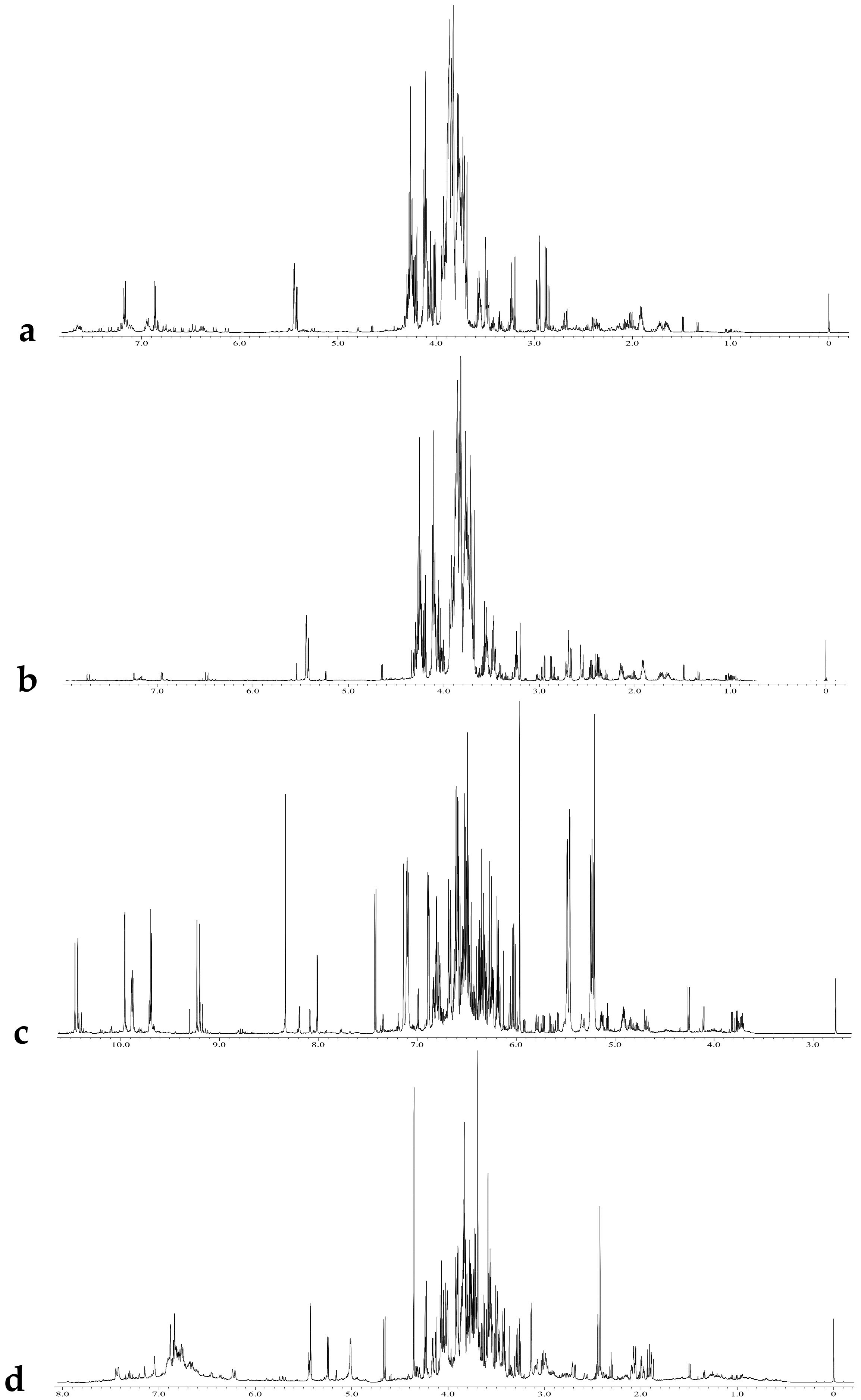

Figure S2. ^{1}H-NMR Spectrum of the hydroalcoholic Bligh-Dyer extract, in 100 mM PBS/D_2O, 0.4 mM TSP. (a) Burdock root, (b) Dandelion root, (c) Dandelion aerial part, (d) Lemon balm aerial part.

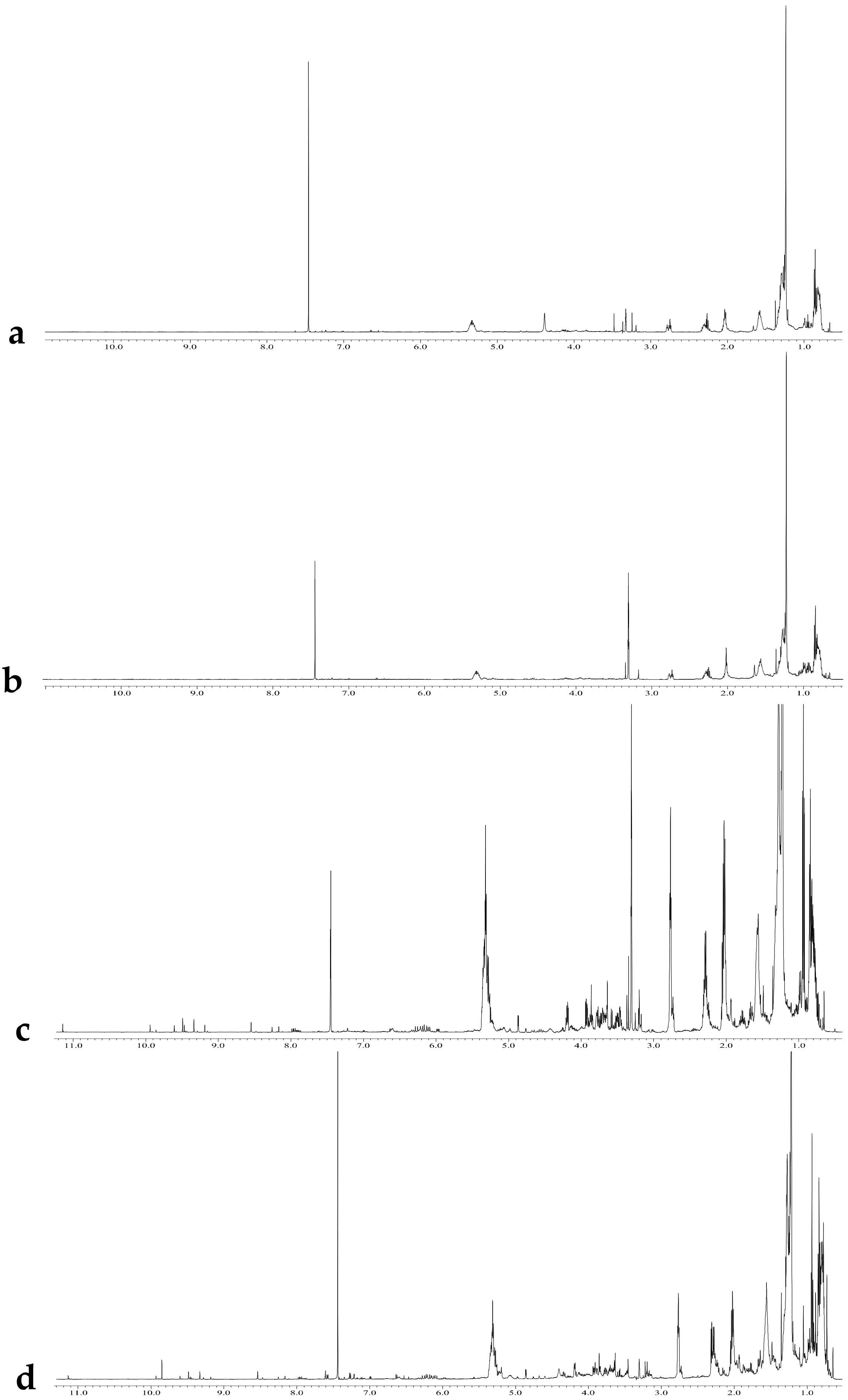

Figure S3. ^{1}H-NMR Spectrum of the organic Bligh-Dyer extract, in $CDCl_3/CD_3OD$ (2:1 v/v) mixture. (a) Burdock root, (b) Dandelion root, (c) Dandelion aerial part, (d) Lemon balm aerial part.

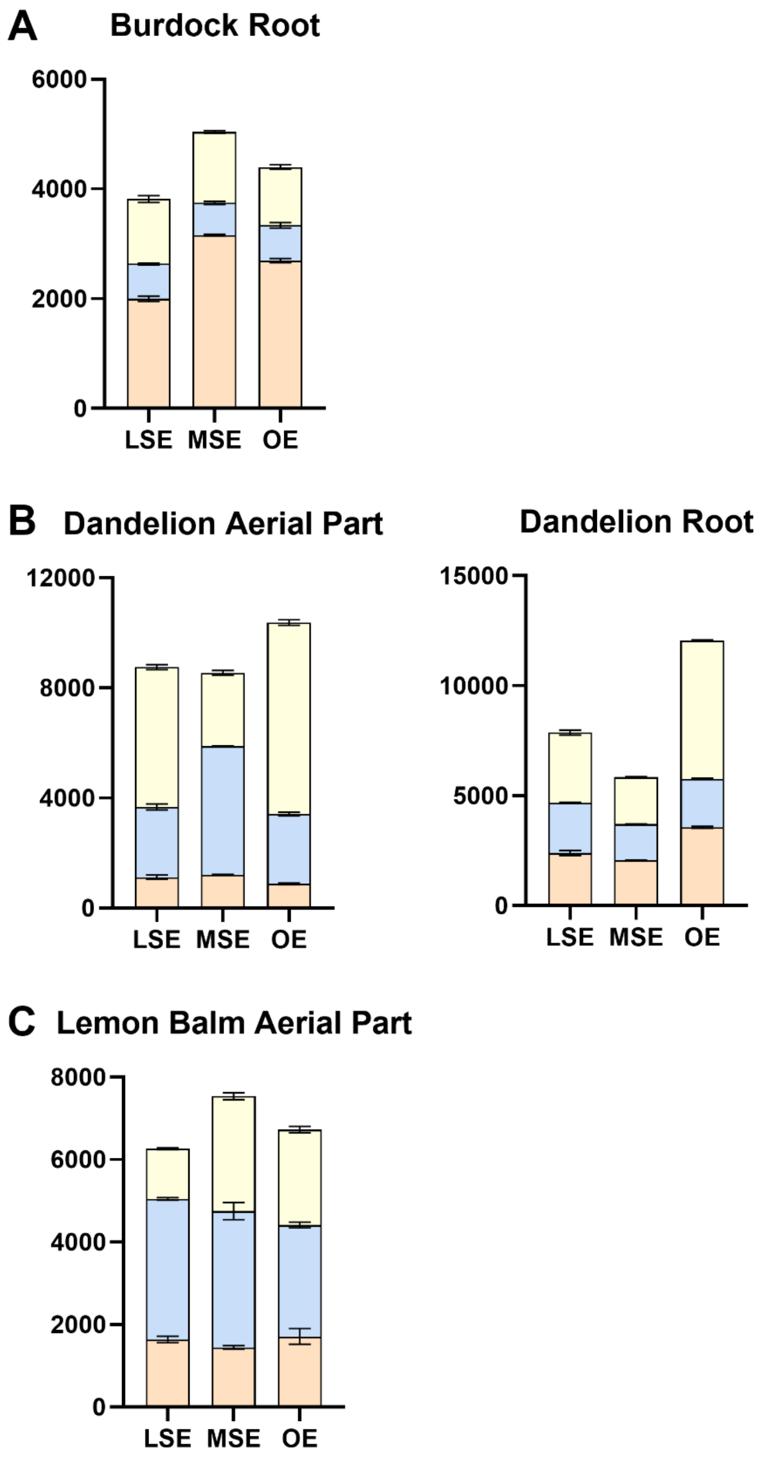

Figure S4. Histograms resulting from the quantitative NMR analysis of main compound total content amino acids (orange), organic acids (light blue) and carbohydrates (yellow) present in the Bligh-Dyer hydroalcoholic extracts of (A) Burdock root, (B) Dandelion aerial part and root, and (C) Lemon Balm in the three ecotypes Land Spontaneous (LSE), Organic (OE), Mountain Spontaneous (MSE). Results, expressed as mg/100 g dried sample, refer to the mean and SD of three replicates.

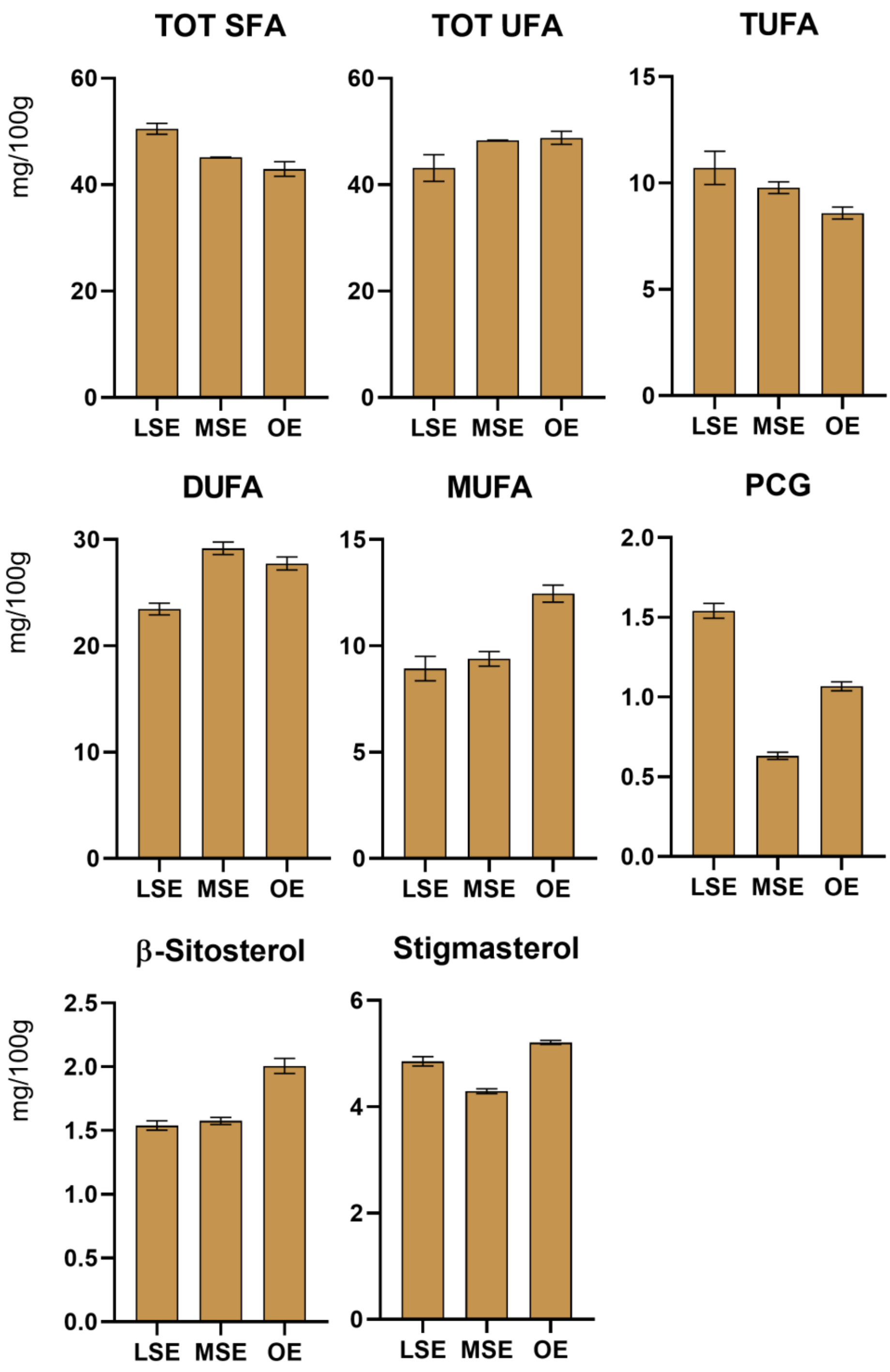

Figure S5. Histograms relative to compounds quantified (mg/100 g of dried sample ± SD) in Bligh-Dyer organic extracts of Burdock in Land Spontaneous (LSE), Mountain Spontaneous (MSE) and Organic (OE).

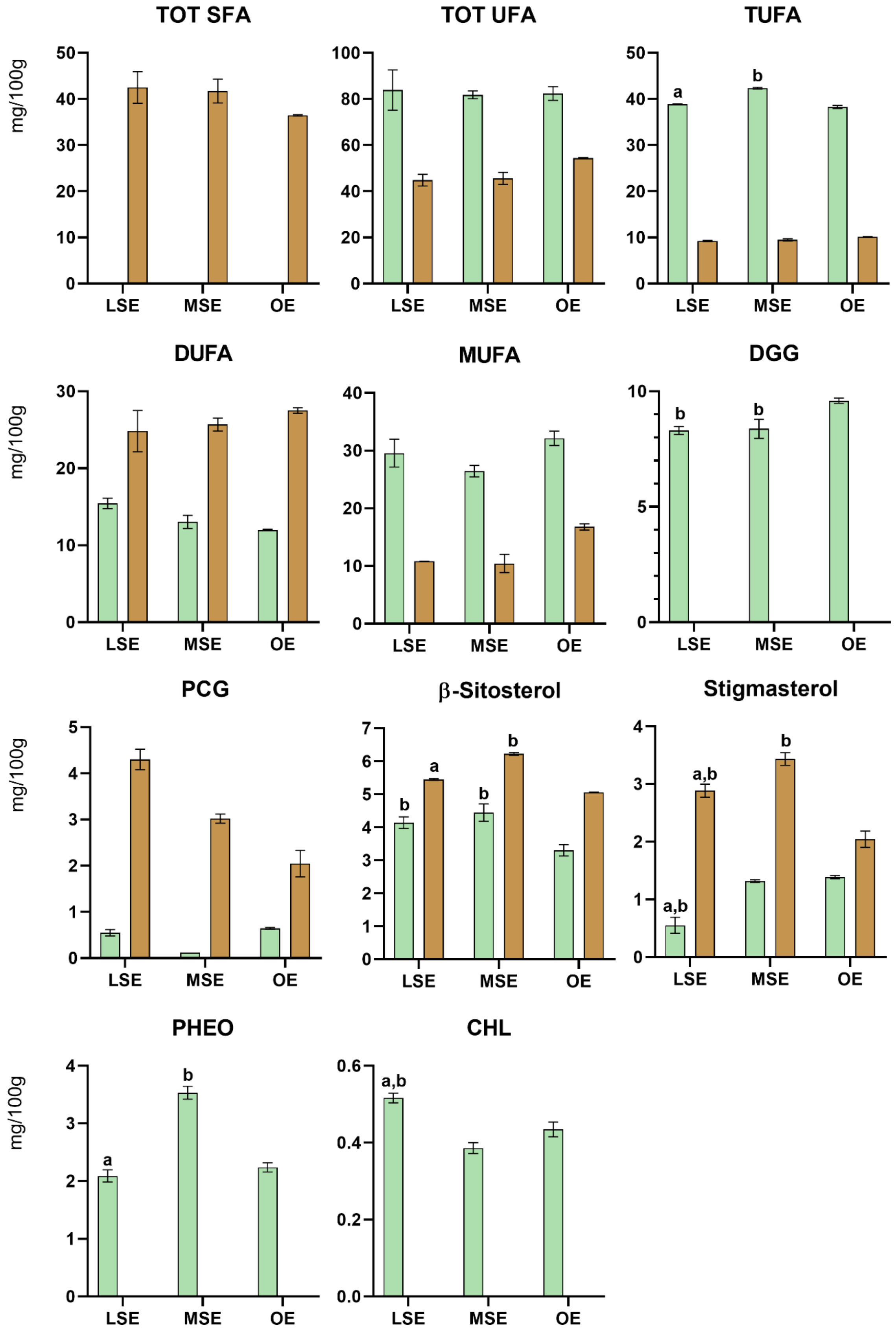

Figure S6. Histograms relative to compounds (mg/100 g dried sample ± SD) in Bligh-Dyer organic extracts of Dandelion aerial part (green) and root (brown) in Land Spontaneous (LSE), Mountain Spontaneous (MSE) and Organic (OE). Two-way ANOVA, followed by Tukey's multiple comparison test, was applied to underline, among ecotypes, significant differences ($p<0.0001$) for each metabolite according to the same plant part: a) vs. MSE, b) vs. OE.

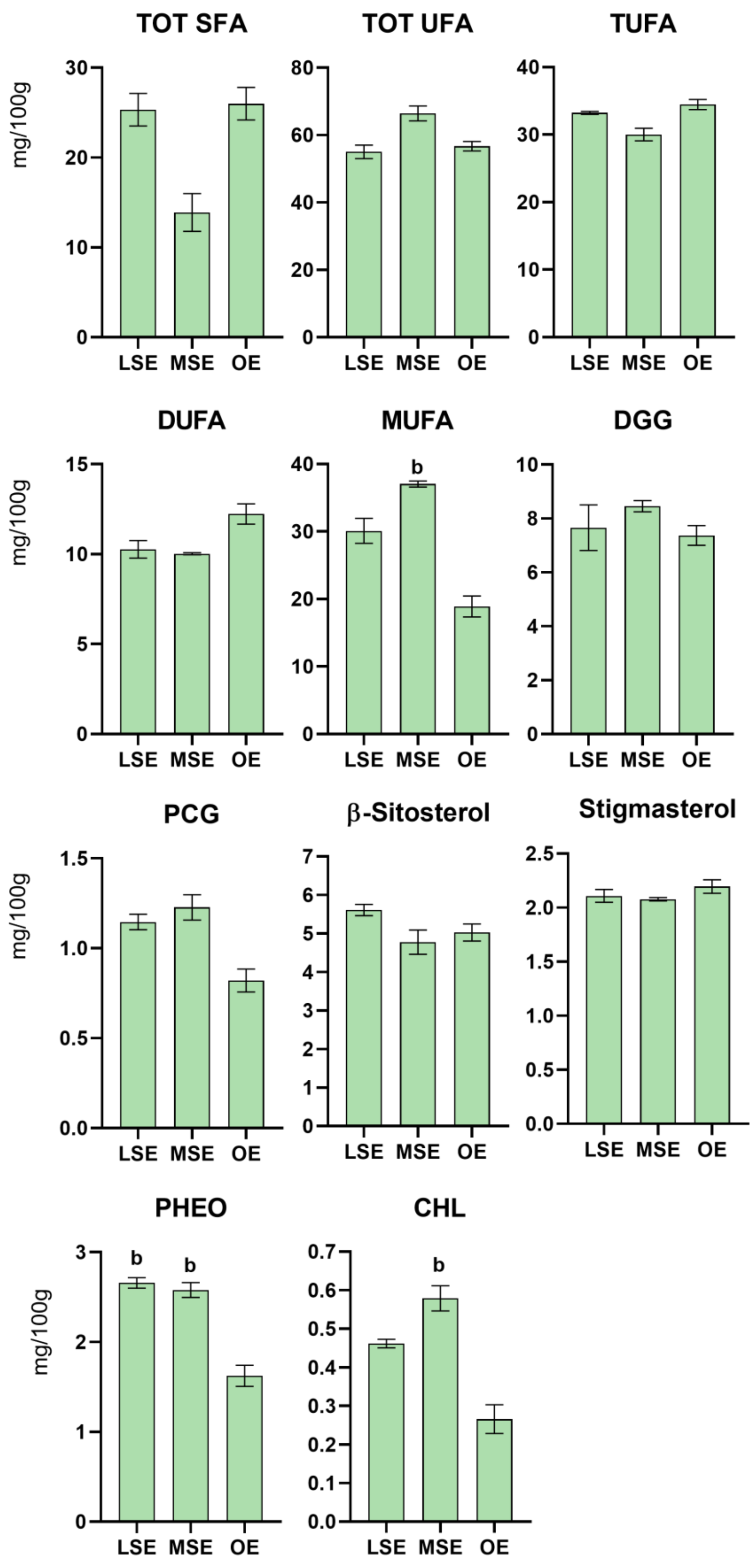

Figure S7. Histograms relative compounds quantified (mg/100 g of dried sample ± SD) present in Bligh-Dyer organic extracts of Lemon balm in Land Spontaneous (LSE), Mountain Spontaneous (MSE) and Organic (OE). One-way ANOVA, followed by Tukey's multiple comparison test, was applied to underline, among ecotypes, significant differences ($p<0.0001$) for each metabolite: a) vs. MSE, b) vs. OE.

Tables

Table S1. Environmental growing conditions in Collepardo and Isola del Liri (Italy).

**Collepardo**	**Jan**	**Feb**	**Mar**	**Apr**	**May**	**Jun**	**Jul**	**Aug**	**Sep**	**Oct**	**Nov**	**Dec**	**Mean**
Temperature [T]	3 °C	3 °C	6 °C	9 °C	14 °C	18 °C	21 °C	21 °C	17 °C	12 °C	7 °C	3 °C	**11 °C**
Precipitation (mm) [P]	49.3	51.4	42.2	42.7	33.2	26.0	18.8	23.3	55.2	74.2	86.9	61.1	**47.0**
Humidity [H]	0 %	0 %	0 %	0 %	0 %	1 %	4 %	8 %	3 %	0 %	0 %	0 %	**1.3 %**
Daylight hours [DH]	9.5	10.6	12.0	13.4	14.6	15.2	14.8	13.8	12.4	11.1	9.8	9.2	-
Sun energy (kWh) [SE]	2.0	2.9	4.2	5.5	6.6	7.4	7.5	6.5	4.9	3.4	2.2	1.7	4.5

**Isola del Liri**	**Jan**	**Feb**	**Mar**	**Apr**	**May**	**Jun**	**Jul**	**Aug**	**Sep**	**Oct**	**Nov**	**Dec**	**Mean**
Temperature [T]	5 °C	6 °C	9 °C	12 °C	16 °C	21 °C	24 °C	24 °C	19 °C	15 °C	10 °C	6 °C	**14 °C**
Precipitation (mm) [P]	55.2	55.0	46.6	43.1	32.7	25.8	19.0	23.4	55.3	73.9	89.3	64.7	**48.7**
Humidity [H]	0 %	0 %	0 %	0 %	4 %	4 %	11 %	18 %	4 %	2 %	0 %	0 %	**3.4%**
Daylight hours [DH]	9.5	10.6	12.0	13.4	14.6	15.2	14.8	13.8	12.4	11.1	9.8	9.2	-
Sun energy (kWh) [SE]	2.0	2.9	4.2	5.5	6.6	7.4	7.4	6.5	4.9	3.4	2.2	1.7	4.5

[T] The average temperature, considering the maximum and minimum for a month.

[P] The average rainfall accumulated during 31 days.

[H] Humidity is based on the comfort level at the dew point (if perspiration will evaporate from the skin, cooling the body).

[DH] The number of hours in which the sun is visible.

[SE] The average amount of shortwave solar energy reaching the ground per unit area.
